# Prevalence of PD-L1 expression is associated with EMAST, density of peritumoral T-cells and recurrence-free survival in operable non-metastatic colorectal cancer

**DOI:** 10.1007/s00262-020-02573-0

**Published:** 2020-04-20

**Authors:** Martin M. Watson, Dordi Lea, Einar Gudlaugsson, Ivar Skaland, Hanne R. Hagland, Kjetil Søreide

**Affiliations:** 1 Gastrointestinal Translational Research Unit, Stavanger University Hospital, Stavanger, Norway; 2 Department of Clinical Medicine, University of Bergen, Bergen, Norway; 3 Department of Pathology, Stavanger University Hospital, Stavanger, Norway; 4 Department of Chemistry, Bioscience and Environmental Engineering, University of Stavanger, Stavanger, Norway; 5 Department of Gastrointestinal Surgery, Stavanger University Hospital, Stavanger, Norway

**Keywords:** Colorectal cancer, EMAST, PD-L1, Immunoscore, Survival, Recurrence

## Abstract

**Introduction:**

Microsatellite instability (MSI) predict response to anti-PD1 immunotherapy in colorectal cancer (CRC). CRCs with MSI have higher infiltration of immune cells related to a better survival. Elevated Microsatellite Alterations at Tetranucleotides (EMAST) is a form of MSI but its association with PD-L1 expression and immune-cell infiltration is not known.

**Methods:**

A consecutive, observational cohort of patients undergoing surgery for CRC. EMAST and clinicopathological characteristics were investigated against PD-L1, as well as CD3 and CD8 expression in the invasive margin or tumour centre (Immunoscore). Difference in survival between groups was assessed by log rank test.

**Results:**

A total of 149 stage I–III CRCs patients, with a median follow up of 60.1 months. Patients with PD-L1+ tumours (7%) were older (median 79 vs 71 years, *p* = 0.045) and had EMAST+ cancers (OR 10.7, 95% CI 2.2–51.4, *p* = 0.001). Recurrence-free survival was longer in cancers with PD-L1+ immune cells (HR 0.35, 95% CI 0.16–0.76, *p* = 0.008, independent of EMAST) and high Immunoscore (HR 0.10, 95% CI 0.01–0.72, *p* = 0.022). Patients expressing PD-L1 in immune cells had longer disease-specific survival (HR 0.28, 95% CI 0.10–0.77, *p* = 0.014).

**Conclusions:**

Higher Immunoscore (CD3/CD8 cells) and expression of tumour PD-L1 is found in CRCs with EMAST. Lymphocytic infiltrate and peritumoral PD-L1 expression have prognostic value in CRC.

**Electronic supplementary material:**

The online version of this article (10.1007/s00262-020-02573-0) contains supplementary material, which is available to authorized users.

## Introduction

Colorectal cancers (CRCs) with deficient mismatch repair (MMR) are often hypermutated, have microsatellite instability (MSI), are associated with improved prognosis and is defined to the ‘immunogenic’ class of consensus molecular subtypes [1]. Notably, MSI is determined by a panel of microsatellite markers, commonly mononucleotides, according to established guidelines [2]. However, an alternative form of MSI is found in tetranucleotide-based microsatellites and labelled Elevated Microsatellite Alterations at Selected Tetranucleotides (EMAST) [3, 4]. Currently, the prognostic value, molecular mechanisms and clinical implications of EMAST are unclear. EMAST was linked in vitro with downregulation of MSH3, a member of MMR specifically implicated with repair of long indels [4]. This proposed mechanism has not been confirmed across patient series, with a previous study from our group refuting an association between MSH3 and EMAST [5]. Prognostic data on EMAST is also scarce. In a previous study, we found that patients with EMAST+ were older, frailer and less likely to have recurrence from CRC [6].

CRCs with MSI are associated with a higher production of neoantigens and consequent immune system activation [7]. The understanding of host immune system and its relevance for cancer control has evolved across several tumour types yet with varying potential for therapeutic intervention and effect on disease trajectory [7]. In colorectal cancer (CRC), data suggest that type and density of immune cells are related to survival and may be used to improve TNM-staging by incorporating an Immunoscore [8, 9]. Hence, the immune cells infiltrating in the tumour microenvironment have a functional role in CRC, although understanding of associated factors related to this peritumoral activation is poor at present.

Cancer immunosurveillance of the adaptive immune system may be disturbed through various mechanisms [10]. One example is the activation of immune checkpoints such as the receptor-ligand complex PD-1/PD-L1 that dampens the immune response and cause T-cell exhaustion [7, 11, 12]. Data suggesting that PD-1 blockade therapy potentially benefits the MMR/MSI subsets of CRCs and other cancers [13–15], introduced immunotherapy for clinical use [16]. However, selection of patients who may benefit and respond is currently uncertain. Further, scarce evidence exists to date on the association of PD-L1 expression and prognosis and survival, both within and outside the predictive subsets of CRC. Data regarding the relationship between EMAST and PD-L1 expression and the associated T-cell infiltration are lacking.

The aim of the present study was thus to describe the prevalence of PD-L1 expression, Immunoscore, their relationship with MSI/EMAST and their relevance towards clinical outcomes in a well-defined, consecutive series of operable CRCs.

## Materials and methods

### Study population and design

Patients were consecutively recruited during the 01/2013–05/2015 period at Stavanger University Hospital (SUH), Norway. Norway has a universal health care coverage for all citizens and the university hospital serves a primary catchment region of about 370,000 inhabitants. With no selection or referral bias in the health care system, the study cohort can be considered as population representative and generalizable to similar regions in Northern Europe.

The present study cohort is part of an ongoing prospective project (ACROBATICC) approved by the regional ethics committee (REK Helse Vest: 2012/742) and registered on clinicaltrials.gov (NCT01762813) [17]. All consecutive patients amenable to curative intent surgery, aged ≥ 18 years of age and who could provide written informed consent were eligible for inclusion into ACROBATICC. This observational cohort study of patients presenting with operable stage I–III disease and is reported according to the STROBE [18] and the REMARK [19] guidelines for biomarker studies.

### Histopathology

All cancers were staged by an experienced pathologist following guidelines published in the 7th edition of the AJCC staging manual [20]. Proximal tumour location is intended as the region between caecum and transverse colon, while distal is intended as the region between the splenic flexure and sigmoid colon.

### EMAST and MSI analysis

Analyses of EMAST and MSI, including primer sequences and PCR conditions, are described previously [21, 22]. Briefly, formalin-fixed paraffin blocks selected by an experienced pathologist were sectioned for DNA extraction. Macrodissection of areas indicated by the pathologist was employed where necessary to enrich for tumour cells. Automated DNA extraction was carried out using AllPrep DNA/RNA FFPE kit (Qiagen, Hilden, Germany) on a QiaCUBE instrument (Qiagen), according to manufacturer’s instructions. Nucleic acid concentration and purity were measured on a NanoDrop 2000 (ThermoFischer scientific, Waltham, USA). Two separate multiplex PCR reactions (one for each MSI and EMAST) were set up for tumour and normal DNA in each patient. TypeIT microsatellite (Qiagen) master mix, together with a blending of 5 × 5′-fluorescently labelled primer pairs was used for each reaction. The primers for MSI were specific for the quasimonomorphic mononucleotides BAT-26, NR-21, NR-24 and NR-27, while the EMAST marker panel consisted of MYCL1, D8S321, D9S242, D20S82, and D20S85. To define a tumour as MSI-H, at least 2/5 markers needed to be unstable in their respective panels.

### Immunohistochemistry

Paraffin sections consecutive to the haematoxylin–eosin (H&E) sections were cut to 2 µm and mounted onto Superfrost Plus slides (Menzel, Braunschweig, Germany). Antigen retrieval and antibody dilution were optimised for each induvial staining. All antibody protocols were optimized before study onset. Paraffin sections consecutive to the haematoxylin–eosin (H&E) sections were cut to 2 µm and mounted onto Superfrost Plus slides (Menzel, Braunschweig, Germany). Slides were incubated at 60 °C for 1 h and then transferred to a Dako Omnis (Dako, Glostrup, Denmark) instrument. CD3 (Dako, Clone F7.2.38) was used at a dilution of 1:75 and visualised by EnVision FLEX, High pH (Dako Omnis) (GV80011-2). CD8 (Dako, Clone C8/144B) was used at a dilution of 1:50 and visualized by EnVision FLEX, High pH (Dako Omnis) (GV80011-2).with EnVision FLEX+ Mouse LINKER (Dako Omnis) (GV82111-2) signal amplification.

EnVision FLEX Antibody Diluent (Dako, K800621-2) was used as diluent. Pre-treatment time was 20 min at 97 °C using EnVision FLEX Target Retrieval Solution, High pH (50 ×) (Dako Omnis) (GV800). Both antibodies were incubated for 20 min. Hematoxylin (Dako Omnis) (GC80811-2) was used as counterstain.

PD-L1 IHC 22C3 pharmDx (Dako SK00621-2) was used strictly according to manufacturer’s recommendation on a Dako Autostainer Link 48 instrument.

### Scoring of PD-L1 expression

**Fig. 1:**
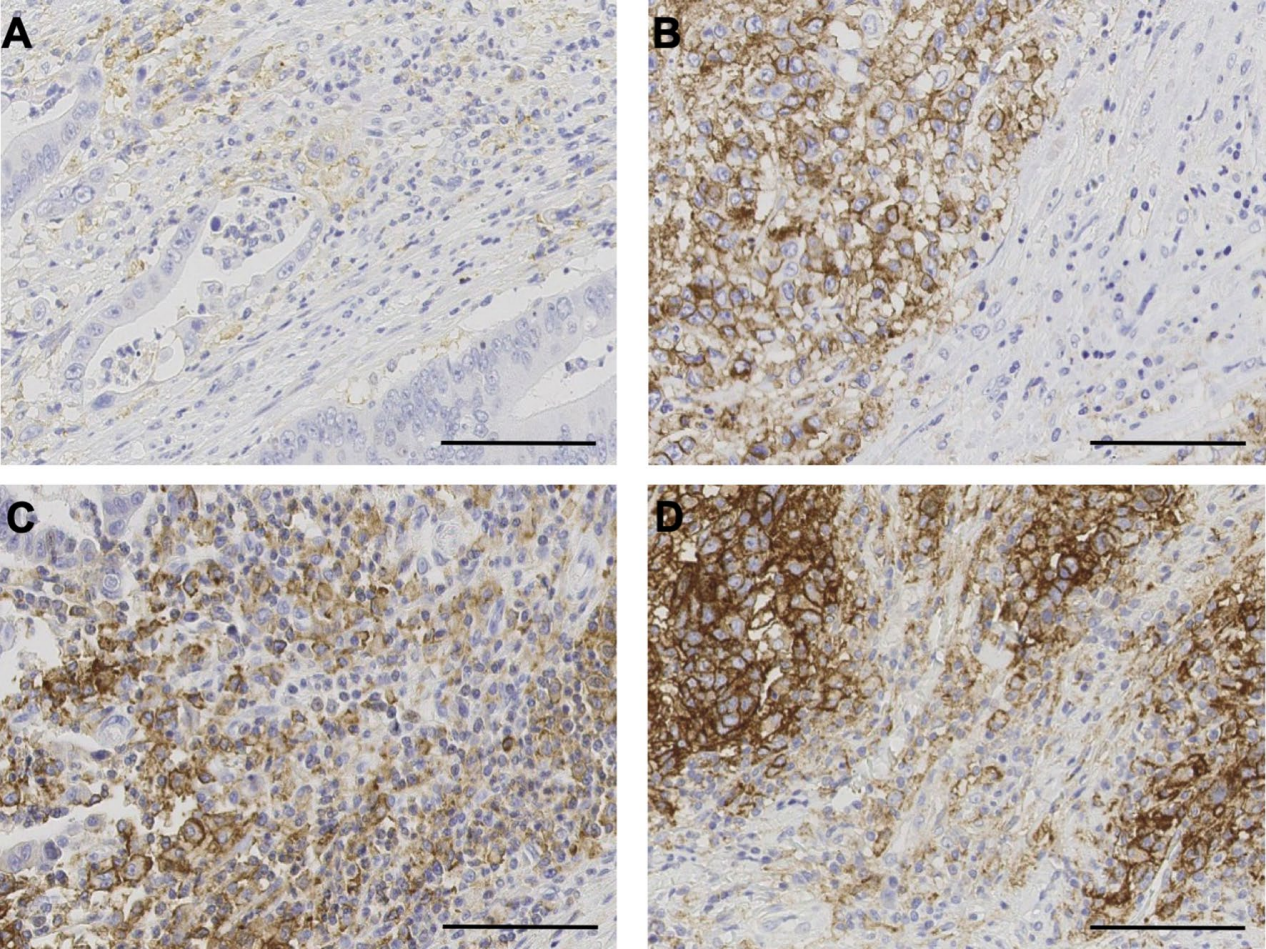
Immunohistochemistry of PD-L1. 20X magnification view of **a** immune PD-L1−/tumour PD-L1−. **b** Immune PD-L1−/tumour PD-L1+. **c** Immune PD-L1+/tumour PD-L1−. **d** Immune PD-L1+ / tumour PD-L1+. Scale bar represents 100 μm

PD-L1 expression (Fig. 1) was assessed independently by two experienced pathologists (DL and EG), blinded to patients’ other characteristics and each other results, on whole sections. Membranous staining was regarded as positive, and staining intensity was not evaluated. PD-L1 in tumour cells was scored as positive or negative using ≥ 5% positive as cut-off, based on previous studies [14, 23].

For PD-L1 expression on peritumoral immune cells, the percentage of positive cells were evaluated in the visually most positive area of 1 mm^2^ in the invasive margins of the tumour on the scanned slides (same area for both pathologists). In cases with > 10% discordance between the pathologists, the slides were reviewed together, until consensus was reached. For expression in less than 10% of the immune cells, discordance of < 5% was accepted.

The cut-off for positive or negative classifications of patients based on PD-L1 expression in peritumoral immune cells, was determined experimentally.

Receiver operator characteristics (ROC) curve analysis was used to determine cut-offs for PD-L1 expression in immune cells, with disease-specific death and disease recurrence as the endpoints. The optimal cut-off point in both ROC curve analysis corresponded to the 25th percentile and was therefore chosen as the discriminant cut-off to dichotomize expression of immune cells PD-L1 into positive/negative.

### Immune scoring of CD3 and CD8 markers

All the sections were scanned at 40 × magnification using Leica SCN400 slide scanner (Leica Microsystems, Wetzlar, Germany) and uploaded onto the image analysis software Visiopharm^®^ (Hoersholm, Denmark). Tumour centre and invasive margin areas were marked manually on whole slide images and the same areas were used for the CD3 and CD8 stained sections. Using Bayesian optimisations [24], an algorithm was developed to identify and label CD3+ and CD8+ T-cells in both regions.

Relative quantification of positive cells was obtained by dividing the Visiopharm-measured area of positive label by the estimation of mean area of a lymphocyte (60 µm^2^), thereby approximating the number of CD3+ and CD8+ T-cells per square millimetres (cells/mm^2^). All the cases were inspected, and unspecific staining and artefacts were manually removed from the analyses where appropriate. For individual CD3/CD8 analysis, patients were assigned either a “low” or “high” score for each individual staining (CD3 and CD8), in each tumour location (tumour centre and invasive margin and IM), using the 75th percentile as a threshold. This created four categories (CD3 and CD8, in tumour centre and invasive margin as either high or low).

The Immunoscore was calculated as described elsewhere [8]. Briefly, the densities (in cells/mm^2^) of CD3+ and CD8+ cells in both tumour centre and invasive margin were first converted into percentiles, and then the mean value of the four percentiles calculated. An Immunoscore of “Low”, “Intermediate” or “High” was then assigned to each patient according to their mean percentile scores, with cut-offs as 0–25%, 25–70%, and 70–100% respectively, as described in [8].

### Collection of clinical data and follow up

Clinical measurements as well as follow up data (cause and date of death, date of recurrence) were retrieved from the electronic patient records. Patients’ surveillance after surgery was according to the national guidelines as an interval-based serum CEA (quarterly) and imaging (e.g. a biannual CT-chest and US liver for the first 3 years, then annually) for up to 5 years after surgery. Colon cancers were usually followed up by general practitioners while rectal cancers were seen by gastrointestinal surgeons in the hospital outpatient clinics. Any suspected recurrence or deviation on imaging were worked up in-hospital and consulted in multidisciplinary team meetings, where applicable. The patients’ electronic health records were queried for any documented events, and follow-up for this study was completed as of 24th September 2019.

### Definition of survival endpoints

Recurrence-free survival (RFS) was defined as time from primary surgery until first clinical evidence (histologically confirmed or image-based) of recurrent disease. Disease-specific survival (DSS) was defined as time from primary surgery and death imputable to CRC. Survival was assessed for overall survival (OS) defined as time from primary surgery to death of any cause.

### Statistical analyses

All statistical tests were done using IBM SPSS Statistics for Windows, Version 25.0 (IBM Corporation, Armonk, NY, USA). Associations between categorical variables were tested with Chi-square (or Fischer’s exact test, where appropriate) method and reported with odds ratios and 95% confidence interval (95% CI). Spearman’s rho or Pearson tests were used for correlations between continuous/ordinal variables, where appropriate. Mann–Whitney *U* test was used to compare differences in continuous or ordinal variables between groups. Inter-coder reliability score for PD-L1 evaluation was estimated using the KALPHA extension for SPSS and expressed as Krippendorff’s alpha (*α*).

The Kaplan–Meier method with log rank comparison of factors was used to investigate survival curves differences between groups and are given as (months difference [95% CI]). Univariable proportional hazards are given in hazard ratios (HR) with 95% CI. All tests were two-tailed and a *p* value < 0.050 considered as statistically significant.

## Results

**Fig. 2:**
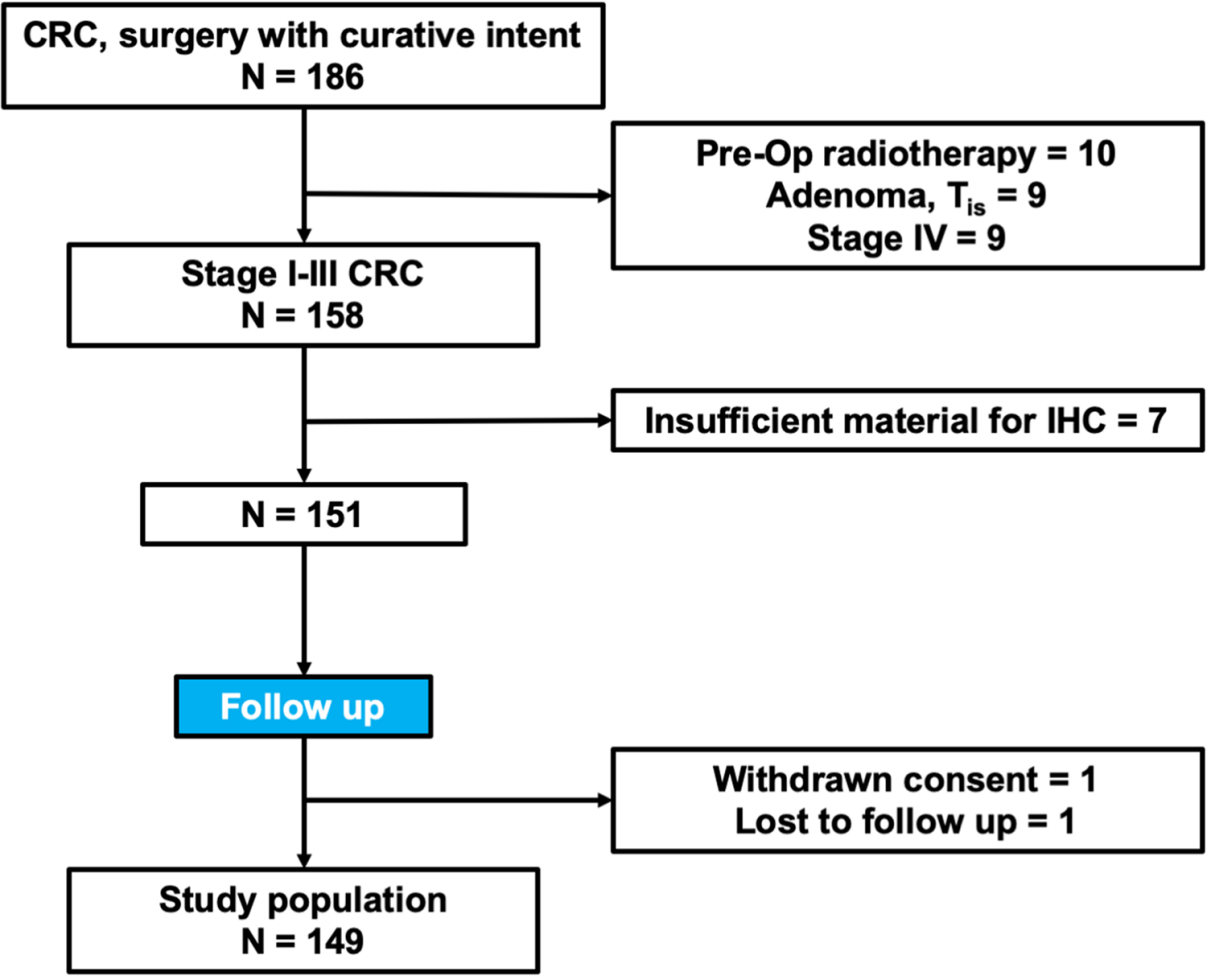
Flowchart of inclusion/exclusion criteria. CRC denotes colorectal cancer; IHC denotes immunohistochemistry

The study cohort included 149 stage I–III CRC patients who underwent surgery with curative intent (Fig. 2). Patients’ descriptive parameters are included in Table 1.

**Table 1:** Variables associated with EMAST status

	Total *n*, (%)	EMAST− *n* = 99 (66)	EMAST+ *n* = 50 (34)	*p*
Age				**< 0.001**
Median (range)	72 (37–92)	70 (37–91)	77.5 (50–92)	
≤ 72	75 (50)	60 (80)	15 (20)	
> 72	74 (50)	39 (53)	35 (47)	
Sex				**< 0.001**
Male	65 (44)	54 (83)	11 (17)	
Female	84 (56)	45 (54)	39 (46)	
Localisation				**< 0.001**
Colon	124 (83)	74 (60)	50 (40)	
Rectum	25 (17)	25 (100)	0 (0)	
Within colon				**< 0.001**
Right	70 (57.5)	25 (36)	45 (64)	
Left	54 (43.5)	49 (90)	5 (10)	
Grade*				**< 0.001**
High	39 (26)	15 (38)	24 (62)	
Low	109 (74)	83 (76)	26 (24)	
Stage				0.234
I	51 (34)	30 (59)	21 (41)	
II	50 (34)	33 (66)	17 (34)	
III	48 (32)	36 (75)	12 (25)	
MSI				**< 0.001**
MSS	105 (70.5)	97 (92)	8 (8)	
MSI-H	44 (29.5)	2 (5)	42 (95)	

*N* = 149

Bold values indicate statistical significance (*P* < 0.050)

*One missing

### PD-L1 expression and EMAST

**Fig. 3:**
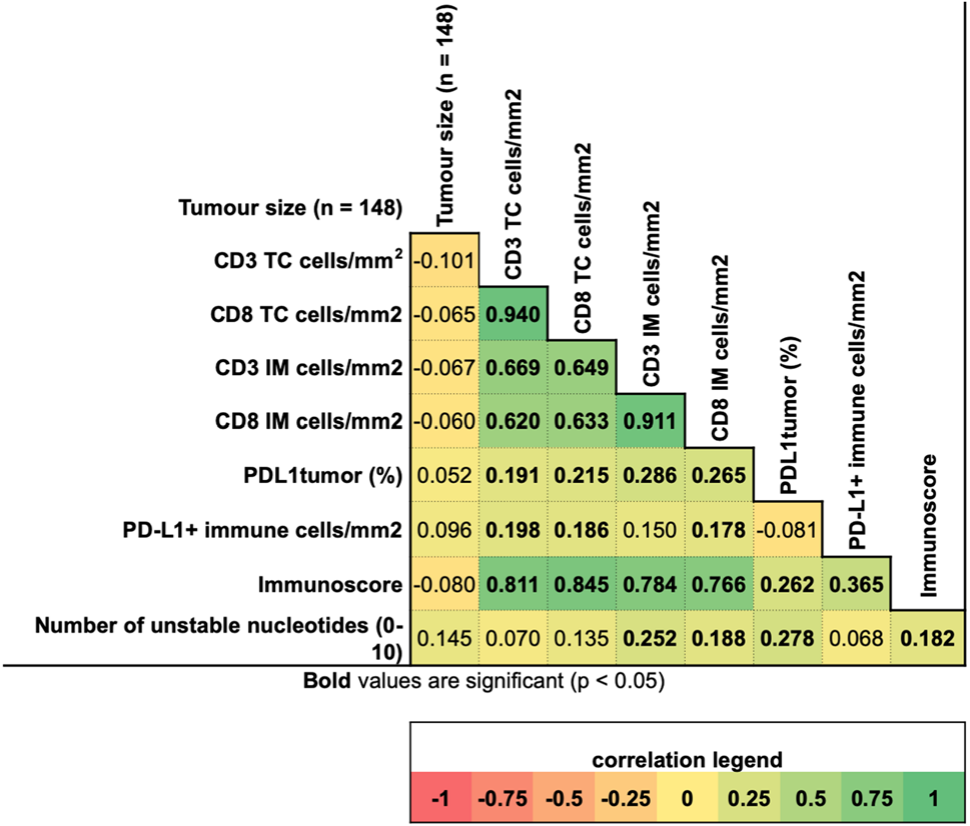
Correlation matrix of immune-related variables. For pure ordinal variables (marked by the notation “cells/mm2”), Pearson correlation coefficient is shown. For all other variable Spearman R-O tests were used. Bold correlation coefficients are significant (*p* < 0.05)

Of the 11 patients classified as PD-L1+ in tumour cells, nine were diagnosed with right-side CRC (82%, no rectum, *p* = 0.111) and were EMAST+ (82%; Table 2). Inter-coder reliability score for PD-L1 expression in tumour cells was high (Krippendorff’s *α* = 0.93; 95% CI 0.83–0.99). A weak correlation was also seen between expression of tumoral PD-L1 and total number of unstable markers analysed for both EMAST and MSI (Fig. 3; *p* = 0.001). A higher number of markers from the two panels combined were indeed unstable in PD-L1+ tumours (median 9/10 vs 1/10 markers, *p* = 0.001), when dichotomised accordingly (Suppl. Table 1). Tumour PD-L1+ patients were significantly older (79 vs 71 years, *p* = 0.045) and had lower preoperative levels of serum albumin (33.6 vs 38.1 g/L, *p* = 0.011) (Suppl. Table 1). All PD-L1+ tumours (11/11, 100%) were in the colon, while none of the 25 rectum tumours scored positive (*p* = 0.212).

**Table 2:** Associations with immune markers and EMAST status

*N* = 149	Total *n*, (%)	EMAST− *n* = 99 (66)	EMAST+ *n* = 50 (34)	OR (95% CI)	*p*
PD-L1 in tumour cells				10.7 (2.2–51.4)	**0.001**
Low	138 (93)	97 (98)	41 (82)		
High	11 (7)	2 (2)	9 (18)		
PD-L1 in immune cells				1.0 (0.5–2.2)	0.973
Low	39 (26)	26 (26)	13 (26)		
High	110 (74)	73 (74)	37 (74)		
Immune cells in tumour centre					
CD3+				2.37 (1.1–5.1)	**0.025**
Low	112 (75)	80 (81)	32 (64)		
High	37 (25)	19 (19)	18 (36)		
CD8+				2.4 (1.1–5.1)	**0.025**
Low	112 (75)	80 (81)	32 (64)		
High	37 (25)	19 (19)	18 (36)		
Immune cells in invasive margin					
CD3+				3.22 (1.5–7.0)	**0.002**
Low	112 (75)	82 (83)	30 (60)		
High	37 (25)	17 (17)	20 (40)		
CD8+				2.4 (1.1–5.1)	**0.025**
Low	112 (75)	80 (81)	32 (64)		
High	37 (25)	19 (19)	18 (36)		
Immunoscore				n.c.	**0.020**
Low	31 (21)	24 (24)	7 (14)		
Interm	79 (53)	56 (57)	23 (46)		
High	39 (26)	19 (19)	20 (40)		

Bold values indicate statistical significance (*P* < 0.050)

In peritumoral infiltrating immune cells, the rate of PD-L1 expression was higher than in tumour cells (Fig. 1). No statistically significant association was found between expression of PD-L1 in immune cells and patients’ age, EMAST status or number of unstable markers (Suppl. Table 1**)**. Again, a significant but small correlation was found between % PD-L1 and CD3/CD8 in immune cells, albeit not in the case of CD3 in the invasive margin. The two ROC analyses for determination of ideal cut-off value of % PD-L1 positive immune cells had AUC = 0.698, *p* = 0.012 with disease-specific death and AUC = 0.648, *p* = 0.018 with disease recurrence as endpoint (data not shown). The 25th percentile cut-off showed no difference in the distribution among colon and rectum cancers, of which 73% and 76% showed PD-L1-positive immune cells, respectively. Inter-coder reliability score for PD-L1 expression in immune cells was relatively high (Krippendorff’s *α* = 0.81; 95% CI 0.72–0.88).

### Immune cell types, Immunoscore and EMAST status

Higher density of CD3+ and CD8+ cells in tumour centre and invasive margins were found in EMAST-positive patients (Table 2).

Immunoscore was distributed into low (*n* = 31, 21%), intermediate (79, 53%) and high (*n* = 39, 26%) categories, respectively. EMAST-positive patients were proportionally more represented in the higher Immunoscore subclasses (Table 2). As expected, Immunoscore correlated strongly with each individual CD3+ and CD8+ tally. A stronger relationship between Immunoscore and % of PD-L1+ in immune (Spearman 0.365, *p* < 0.001) rather than in tumour cells (0.262, *p* = 0.001) was found (Fig. 3).

Tumours with PD-L1+ tumours had significantly higher counts of CD3 and CD8 in the invasive margin, as well as CD8, but not CD3 in the tumour centre (Suppl. Table 1).

Patients with PD-L1+ immune cells had significantly higher counts of CD3 and CD8 in both the invasive margin and tumour centre (Suppl. Table 1). Both immune PD-L1+ (*p* < 0.001) and tumour PD-L1+ (*p* = 0.037) patients were significantly associated with a high Immunoscore.

### Risk of recurrence and recurrence-free survival

During the follow up period, a total of 26 (17.4%) patients experienced recurrent disease. Eight recurrences (31%) were in the liver, eight in the lungs, 7 (27%) were local recurrences, and one (4%) each for bone, peritoneum and brain.

Generally, higher numbers of infiltrating lymphocytes correlated with lower risk of disease recurrences (Suppl. Figure 1).

**Fig. 4:**
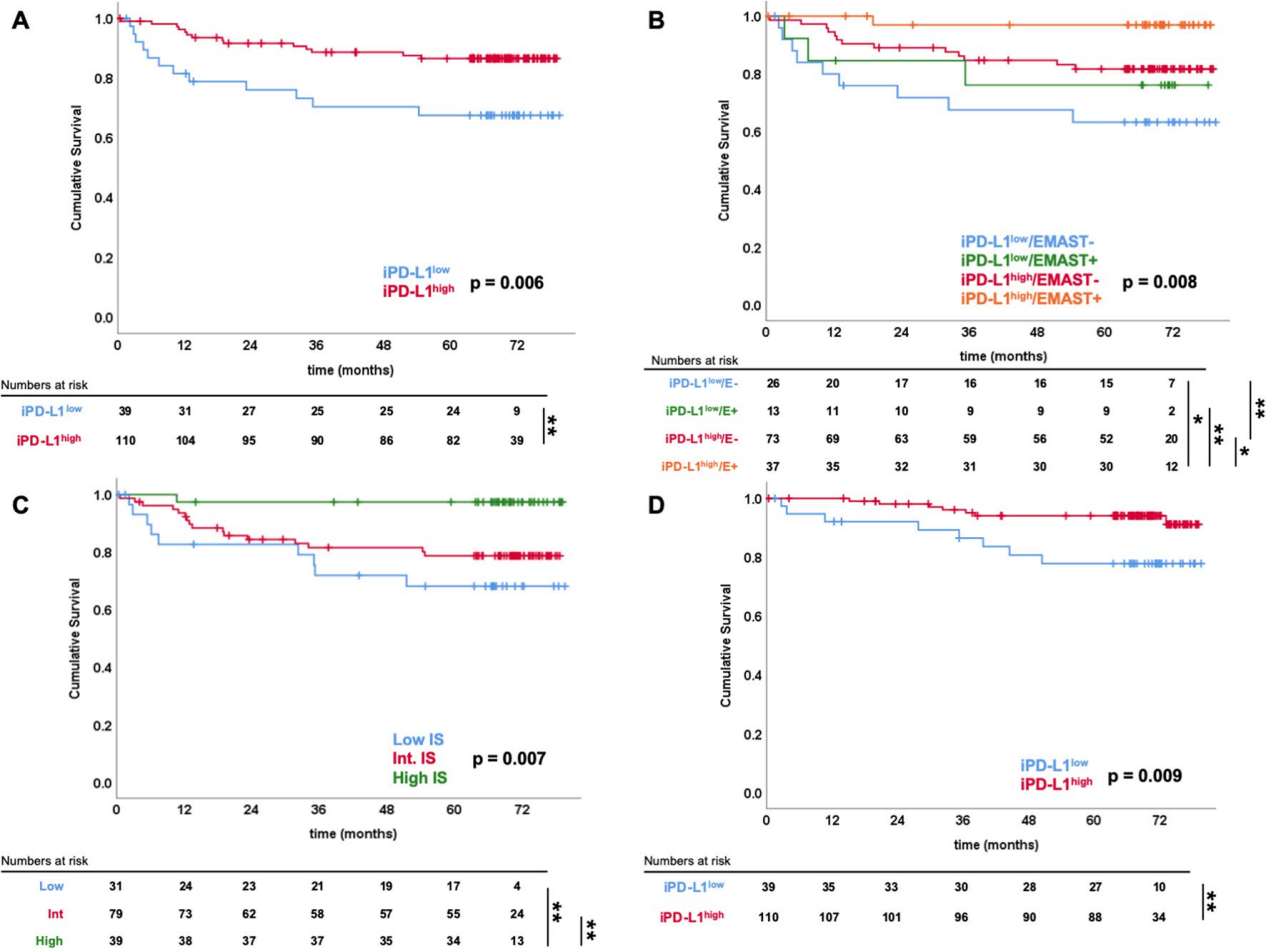
Survival analyses comparing prognostic groups. Kaplan-Meier analysis of **a** recurrence-free survival (RFS) of immune PD-L1+/− groups. **b** RFS of immune PD-L1+/− groups, stratified for EMAST status. **c** RFS of Immunoscore groups (low-intermediate-high). **d** Disease-specific survival (DSS) of immune PD-L1+/− groups

A significant difference was found between the categories of the Immunoscore for RFS (Fig. 4; Table 3). No significant association between tumour PD-L1 and rate (*p* = 0.690) or time to recurrence (*p* = 0.520) were recorded. Of the patients with negative immune-PD-L1 expression, 12 (31%) presented with recurrent disease patients against 14 (13%) of those with immune-PD-L1+ . Patients with PD-L1+ immune cells had longer estimated RFS (72 [68–75] vs 59 [49–69] months, *p* = 0.006) than immune-PD-L1− patients, independently of EMAST status (*p* = 0.041 and 0.021 in EMAST− and EMAST+ cases, respectively) (Fig. 4).

**Table 3:** Univariate analyses for survival

Term	HR	95% CI	*p*
Recurrence-free survival (26/149)*			
PD-L1 in immune cells	0.35	0.16–0.76	**0.008**
PD-L1 in tumour cells	0.53	0.07–3.87	0.527
Immunoscore (int. + low vs high)	0.10	0.01–0.72	**0.022**
pN (N0 vs N+)	6.94	2.91–16.52	**< 0.001**
EMAST	0.35	0.12–1.02	0.054
Disease-specific survival (15/149)*			
PD-L1 in immune cells	0.28	0.10–0.77	**0.014**
PD-L1 in tumour cells	0.98	0.13–7.44	0.982
Immunoscore (int. + low vs high)	0.03	0.00–3.37	0.145
pN (N0 vs N+)	229.76	1.44–36788.19	**0.036**
EMAST	0.51	0.14–1.79	0.289
Overall survival (35/149)*			
PD-L1 in immune cells	0.61	0.30–1.23	0.165
PD-L1 in tumour cells	1.78	0.63–5.05	0.277
Immunoscore (int. + low vs high)	0.42	0.16–1.08	0.073
pN (N0 vs N+)	3.17	1.62–6.19	**0.001**
EMAST	1.04	0.52–2.09	0.915

Bold values indicate statistical significance (*P* < 0.050)

*Numbers in parentheses are events/total number of cases

### Overall and disease-specific survival

At the time of final follow-up, a total of 35 (23%) patients had died. Of those, 15 (43%) were CRC-related deaths. Median follow up length was 68.8 months (range 0.4–79.6) from primary surgery to death or right-censoring.

Only nodal status (pN0 vs pN+ or stage I–II vs stage III) was associated with OS in univariate analyses (Table 3). When stratified according to the three Immunoscore levels, a high Immunoscore had significantly longer overall survival than low (74 vs 60 [50–70] months, *p* = 0.008), but not intermediate (74 [69–79] vs 68 [63–72] months, *p* = 0.192). No difference was noted in survival time when patients were divided according to tumour PD-L1 expression, whilst patients with a higher PD-L1 proportion in immune cells had longer DSS (log rank *p* = 0.009; Fig. 4; Table 3). When stratified for EMAST status, patients with PD-L1+ in immune cells had better DSS in the EMAST-negative group (log rank *p* = 0.033) but not in the EMAST+ group (log rank *p* = 0.107).

## Discussion

In the current study, CRC having EMAST correlated with a higher count of intra- and peritumoral CD3+ and CD8+ T-cells and a higher Immunoscore compared to CRC cancers with no EMAST. Also, PD-L1 expression occurred both in immune cells and in tumour cells in CRCs, specifically those with EMAST and MSI.

While the patterns of expression in tissue does not directly translate into functional ability, there are several observations we would point out as being of interest.

First, expression of PD-L1 showed a dual role according to its localisation in this study. Tumour cell-confined PD-L1 correlated with EMAST and generally increasing degree of MSI, while immune cells PD-L1 did not. EMAST independently correlated with a generally higher immunogenicity, with higher levels of CD3+ , CD8+ and PD-L1+ in tumour cells. This is generally in accordance with the relationship between MSI, high mutational burden, generation of tumour neoantigens, and activation of the immune system [7, 25]. Notably, one study previously reported an association with EMAST and CD8+ but not CD4+ T-cells infiltration in tumour [26]. In a previous report, a link between EMAST and older age and a frailer phenotype in patients with EMAST positive cancers was found [6]. These observations, pertinent to a cumulative increase in genetic abnormalities (e.g. EMAST, MSI, mutation burden) during physiological and cellular senescence, may weigh in on the picture of a neoantigen-rich tumour microenvironment. Of note, while the distribution of immune cells expressing PD-L1 seemed comparable between colon and rectum cancers, PD-L1-expressing tumours were exclusively found in colon cancers. This may further confirm a relationship between PD-L1 and instability at microsatellites, as both MSI and EMAST are more prevalent in the colon.

A direct relationship between high Immunoscore and high PD-L1 expression in both tumour and immune cells was also shown in the present study. Tumours with low counts for CD3+ and CD8+ cells are associated with less overall (tumour/immune) PD-L1 expression. PD-L1 was here found to be rarely expressed in tumour cells, and strictly connected to EMAST status, while more diffuse in infiltrating immune cells. This is concordant with recent reports placing tumour PD-L1 rates generally under 15–20% of CRCs, and immune PD-L1 consistently higher [27–32]. These observations may suggest that induction of PD-L1 is regulated by different pathways in immune and tumour cells. On one side, EMAST (as MSI) tumours, due to their higher load of tumour neoantigens are possibly subject to a more vigorous cytotoxic immune response, and endogenously expressing PD-L1 to counteract it. In non-EMAST tumours with high Immunoscore, otherwise, modulation of immune response is achieved by expression of PD-L1 on immune cells, in a mechanism also referred to as adaptive immune resistance [33, 34]. Finally, tumours having both low Immunoscore and PD-L1 expression on immune cells lack an immune reaction in the tumour microenvironment and present with higher rate of recurrences, sooner.

Prognostically, only expression of PD-L1 in the peritumoral cells proved discriminant in both rate of- and time to recurrences, as well as for disease-specific survival. In terms of RFS, the association was independent of EMAST status and comparable to that of Immunoscore, suggesting that immune expression of PD-L1 contributes to the protective effect of tumour immunosurveillance. Tumour PD-L1 was not associated with any of the survival endpoints examined. In contrast to Immunoscore, there is scarce data on the prognostic significance of PD-L1 expression in CRC. The relationship between tumour-expressed PD-L1 and tumour-infiltrating lymphocytes is being investigated in multiple cancers [35, 36]. The focus is however usually on tumour-expressed PD-L1, because of its predictive value for immunotherapy, while there is discordance on its prognostic role [32, 37, 38]. PD-L1 positivity on peritumoral immune cells, on the other hand, is generally a sign of an active immune response and thus associated with improved survival [31, 32, 39, 40].

A limitation of the present study is the limited size of the cohort, with only 11 patients scoring positive for tumour PD-L1, therefore limiting the statistical power. However, the idea of modern personalized medicine is to identify particular subgroups with potential for refined therapy. Prevalence of tumour PD-L1 in MSI CRCs and the low (15–20%) incidence of the subgroup, warrant expansion of the cohort in order to investigate the findings in larger cohorts and refined sub populations. A further limit is the cut-off determination for immune PD-L1 expression. Derived from ROC analysis for recurrence and disease-specific death, which are time-dependent variable, this method may only apply to the present cohort, in the elapsed follow up time. Indeed, a range of methods of PD-L1 scoring and subgrouping are described in the literature, including variation in antibodies used, without a generalised consensus. In the present study, the cut-off value used for PD-L1 expression in tumour cells (5%) was based on previous studies [14, 23, 29], including original anti-PD-1 immunotherapy clinical trials.

The current study correlates PD-L1 expression in tumour cells with EMAST. Moreover, the findings add to the mounting data on PD-L1 expression in peritumoral immune infiltrate and Immunoscore as prognostic factors in CRC. Finally, this study supports the differentiation between tumour- and immune cell expression of PD-L1 as representative of two distinct mechanisms of immune resistance.

### Electronic supplementary material

Below is the link to the electronic supplementary material.

Supplementary file1 (DOCX 20 kb)

Supplementary file2 (TIF 378 kb)

## Abbreviations

CRC: Colorectal cancers
EMAST: Elevated Microsatellite Alterations at Selected Tetranucleotides
MSI: Microsatellite instability
