# Supplementary material for: Prevalence of PD-L1 expression is associated with EMAST, density of peritumoral T-cells and recurrence-free survival in operable non-metastatic colorectal cancer
Source: Cancer Immunol Immunother. 2020 Apr 20;69(8):1627–37. doi: 10.1007/s00262-020-02573-0 (PMC7347699; doi:10.1007/s00262-020-02573-0)
Supplement: Supplementary file 1 — Supplementary file1 (DOCX 20 kb) [file 262_2020_2573_MOESM1_ESM.docx]

SUPPLEMENTARY TABLE 1. Differences in tumour and immune infiltrate PD-L1 expression.

|  | Median (IQR) | Tumour cell PD-L1 expression | | | Immune cell PD-L1 expression | | |
| --- | --- | --- | --- | --- | --- | --- | --- |
|  |  | *low* | *high* | p | *low* | *high* | p |
| **Age** | | | | |  |  |  |
|  | 72  (17) | 71  (17) | 79  (19) | **0.045** | 73  (19) | 71  (15) | 0.842 |
| **# unstable microsatellite markers** | | | | |  |  |  |
|  | 1  (8) | 1  (5) | 9  (8) | **0.001** | 1  (6) | 1  (8) | 0.797 |
| **Serum albumin** | | | | |  |  |  |
|  | 38  (5) | 38  (4) | 34  (4) | **0.038** | 38  (6) | 38  (5) | 0.535 |
| **Tumour centre CD3+** | | | | |  |  |  |
|  | 373  (474) | 341  (424) | 840  (984) | 0.127 | 187  (210) | 451  (592) | **<0.001** |
| **Tumour centre CD8+** | | | | |  |  |  |
|  | 202  (298) | 199  (262) | 545  (658) | **0.024** | 112  (101) | 245  (372) | **<0.001** |
| **Invasive margin CD3+** | | | | |  |  |  |
|  | 827  (873) | 801  (815) | 1538  (1016) | **0.010** | 453  (536) | 919  (786) | **<0.001** |
| **Invasive margin CD8+** | | | | |  |  |  |
|  | 518  (617) | 488  (544) | 1106  (638) | **0.014** | 277  (277) | 593  (619) | **<0.001** |

Values are given as median and (IQR). IQR: interquartile range.
