# Supplementary figures and images for: Prevalence of PD-L1 expression is associated with EMAST, density of peritumoral T-cells and recurrence-free survival in operable non-metastatic colorectal cancer
Source: Cancer Immunol Immunother. 2020 Apr 20;69(8):1627–37. doi: 10.1007/s00262-020-02573-0 (PMC7347699; doi:10.1007/s00262-020-02573-0)

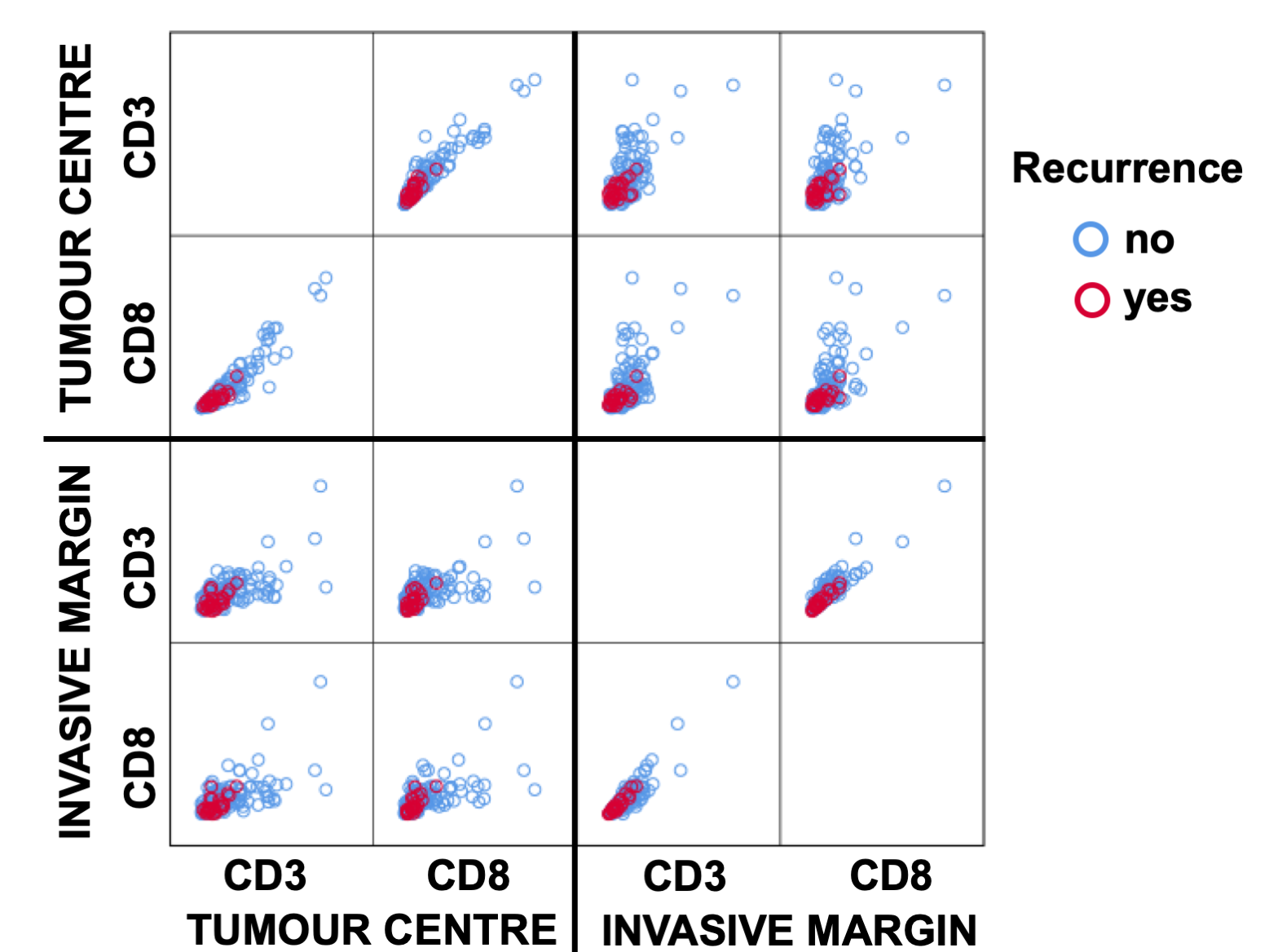

Supplement: Supplementary file 2 — Supplementary file2 (TIF 378 kb) [file 262_2020_2573_MOESM2_ESM.tif]
